# Modeling the rhythmic complexity of professional drumming with an oscillation-driven reservoir computer

**DOI:** 10.1007/s11571-026-10512-5

**Published:** 2026-07-16

**Authors:** Yuji Kawai, Shinya Fujii, Minoru Asada

**Affiliations:** 1 Symbiotic Intelligent Systems Research Center, Institute for Open and Transdisciplinary Research Initiatives, the University of Osaka, 1-1 Yamadaoka, Suita, Osaka 565-0871 Japan; 2 Faculty of Environment and Information Studies, Keio University, 5322 Endo, Fujisawa, Kanagawa 252-0882 Japan; 3 International Professional University of Technology in Osaka, 3-3-1 Umeda, Kita-ku, Osaka, 530-0001 Japan; 4 Academy of Emerging Sciences, Chubu University, 1200 Matsumoto-cho, Kasugai, Aichi 487-8501 Japan

**Keywords:** Reservoir computing, Echo state networks, Microtiming, Rhythm generation, Drumming

## Abstract

Musical performances, particularly in drumming, are characterized not only by their structured rhythmic patterns but also by the subtle variations in timing and amplitude series that create expressive complexity. This study proposes a neural-inspired computational model to investigate how the brain might learn and internalize such complex rhythms. Inspired by the established roles of the cerebellum and basal ganglia in production of rhythms and timings, we utilize an oscillation-driven reservoir computer, a recurrent neural network model for temporal learning, to simulate the generation of human-like expressive drumming performances. First, the model was trained to replicate Jeff Porcaro’s distinctive hi-hat patterns. Analyses revealed that the outputs of the model incorporating high-frequency oscillators ([50, 100] Hz), closely matched the original drumming, reproducing its characteristic fluctuations and patterns in inter-beat timings (microtiming) and amplitudes. Next, the model was trained to generate multidimensional drum kit performances for various genres (funk, jazz, samba, and rock). The model’s outputs exhibited timing deviation and audio features characteristic of the original performances. Our findings demonstrate that oscillation-driven reservoir computing can replicate the rhythmic complexity of professional drumming, suggesting it as a potential computational principle for motor timing and rhythm generation. This approach provides a powerful framework for understanding how the brain generates and processes intricate rhythmic patterns.

**Supplementary Information:**

The online version contains supplementary material available at 10.1007/s11571-026-10512-5.

## Introduction

While a substantial body of research has explored the mechanisms of rhythm perception (Grahn 2012; London 2012; Patel and Iversen 2014; Large et al. 2023), how humans learn and generate complex rhythms remains poorly understood. This generative ability allows us to form internal representations of rhythmic structures, enabling the creation of novel yet stylistically coherent musical rhythms that go beyond simple imitation (Povel 1981). Professional drumming exemplifies this complexity, incorporating subtle timing fluctuations and rich acoustic features.

The performances of professional musicians are characterized by subtle fluctuations in both beat timing (i.e., temporal variations) and velocity (i.e., intensity variations) (Iyer 2002; Fujii et al. 2011; Räsänen et al. 2015; Câmara et al. 2020), which create pleasurable emotional and behavioral responses (Nelias et al. 2022; Kilchenmann and Senn 2015). The temporal aspect of these fluctuations is defined as a deviation from a perfectly isochronous grid of beats, known as the “pulse.” Such a slight deviation from this pulse is termed “microtiming,” usually on a scale of less than 50 ms (Iyer 1998; Butterfield 2006). Räsänen et al. (2015) extracted Jeff Porcaro’s hi-hat playing sound from Michael McDonald’s “I Keep Forgettin’ ” (Mcdonald et al. 1982) and analyzed the hi-hat amplitudes and inter-beat intervals, i.e., time intervals between percussion strokes or hits. The timing intervals drift from the 16th note pattern and follow a Gaussian-type distribution, indicating expressive microtiming. Furthermore, detrended fluctuation analysis (DFA) (Peng et al. 1995) revealed that, like 1/*f* fluctuations, the fluctuations of the timing intervals and amplitudes have persistent correlations over long time scales, i.e., data points separated by long time intervals are not independent but exhibit correlated dependence. However, it is debated whether such microtiming induces a sense of groove (a pleasurable desire to move in response to music (Madison 2006; Madison et al. 2011; Janata et al. 2012; Witek et al. 2014; Madison and Sioros 2014)) in listeners (Etani et al. 2024).

In addition to microtiming, other audio features related to a groove sensation have also been investigated. Madison et al. (2011) found that beat salience (a measure of rhythmic periodicity) and event density (a measure of the variability in the event onset velocity signal) correlate with groove ratings. Stupacher et al. (2016) corroborated Madison et al. (2011)’s result and reported that audio signal intensity, its variability, and attack slope of onsets increase groove ratings.

There is considerable evidence that auditory-motor brain regions activate when listening to music and rhythm (Zatorre et al. 2007; Grahn and Rowe 2009; Teki et al. 2012; Grahn 2012; Fujii and Wan 2014; Merchant et al. 1664; Kasdan et al. 2022; Etani et al. 2024). Kasdan et al. (2022) conducted a meta-analysis of 30 functional magnetic resonance imaging studies that investigated musical rhythm processing. They revealed a large network involving the auditory and motor regions, including the bilateral superior temporal cortices, supplementary motor area, basal ganglia, and cerebellum. In particular, cerebellar activity is greater when the rhythm is more complex, such as in the case of syncopated rhythms. It has been suggested that there is a close relationship between the perception and production of rhythms and that there is overlapped neural circuits (Fujii and Wan 2014; Konoike and Nakamura 2020). The subcortical areas, basal ganglia, and cerebellum respond to auditory time perception and production (Mayville et al. 2002; Teki et al. 2012; Fujii and Wan 2014), and patients with focal lesions in these areas show reduced tracking at the beat frequency (Nozaradan et al. 2017). Furthermore, it has been reported that musical training induces plastic changes in the cortical auditory-motor regions, as well as in the cerebellum and basal ganglia (Penhune 2019). From a broader perspective, these areas play important roles in motor learning and control (Jueptner and Weiller 1998; Doya 2000; Ito 2000; Groenewegen 2003). The basal ganglia perform reward-based learning with dopaminergic modifications while the cerebellum learns by adjusting its connections (long-term depression: LTD) based on feedback (error signals) from climbing fibers. These signals help fine-tune motor skills and coordination (Doya 2000). Through these learning mechanisms, these regions can also learn motor timing (Buhusi and Meck 2005; Grondin 2010; Coull et al. 2011). Although the functional segregation of the cerebellum and basal ganglia in timing perception has been debated (Dreher and Grafman 2002; Teki et al. 2011, 2012; Kameda et al. 2023), we believe that these regions share computational principles that underlie the learning of timing and rhythms.

**Fig. 1:**
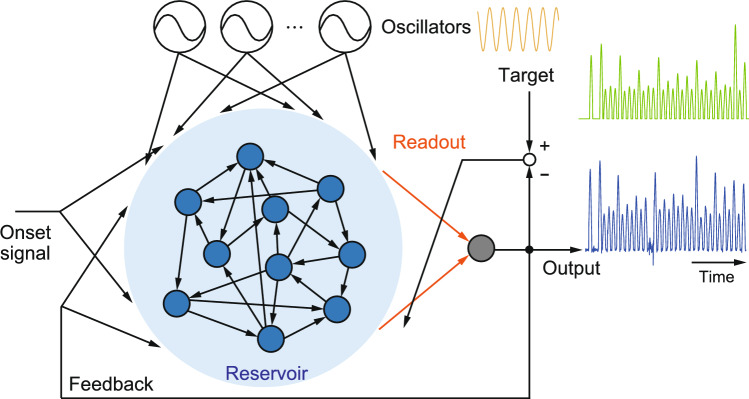
Oscillation-driven reservoir computing. Sinusoidal oscillators drive a reservoir, which is a randomly connected recurrent neural network with fixed connection weights. The output is given as a linear summation of the reservoir states (readout, shown as orange arrows), which is fed back into the reservoir. Only the readout weights are modulated using recursive least-squares. Adapted from Kawai et al. (2025)

Reservoir computing, a type of recurrent neural network (RNN), has been used to model the cerebellum (Yamazaki and Tanaka 2007; Rössert et al. 2015; Tokuda et al. 2021) and basal ganglia (striatum) (Dominey 1995; Hinaut and Dominey 2013; Kawai and Asada 2023; Baladron et al. 2023). The architecture of the model is illustrated in Fig. 1. Standard reservoir computing consists of a fixed randomly connected RNN, termed a reservoir (depicted as the blue network in Fig. 1) (Jaeger and The 2001; Jaeger and Haas 2004). The input to the reservoir network induces complex neural dynamics, and its network states are integrated using a weighted sum readout (indicated by the orange arrows in Fig. 1). The readout weights are modified to obtain the desired output, for which the least-squares method is often used. In the cerebellar models (Yamazaki and Tanaka 2007; Rössert et al. 2015; Tokuda et al. 2021), cortical inputs are conveyed via mossy fibers to a vast population of granule cells. This network of excitatory granule cells and inhibitory Golgi cells as a “reservoir,” generates complex, high-dimensional activity patterns from the inputs. These patterns are then “read out” by Purkinje cells, which receive input from the granule cells’ axons (parallel fibers). The learning process is implemented as synaptic plasticity (LTD) at these parallel fiber–Purkinje cell synapses, adjusting the final output sent to the deep cerebellar nuclei. in the basal ganglia models (Dominey 1995; Hinaut and Dominey 2013; Kawai and Asada 2023; Baladron et al. 2023), the striatum is the primary input structure, receiving projections from the cerebral cortex. The network of medium spiny neurons within the striatum can be seen as a reservoir processing these cortical signals. “Readout training” in this system corresponds to the dopamine-dependent plasticity at the cortico-striatal synapses. Dopamine is thought to signal reward prediction error, and it modulates the synaptic weights to reinforce connections that lead to rewarding actions.

Reservoir computing, which enables temporal information processing based on neural dynamics, has been utilized as a model to explain motor timing learning (Laje and Buonomano 2013; Vincent-Lamarre et al. 2016; Kawai et al. 2023b, a; Kawai and Asada 2023; Kawai et al. 2025). The challenge of sustaining neural activity is fundamental to timing tasks, as exemplified by the classic case of trace eyeblink conditioning (Solomon et al. 1986; Steinmetz et al. 1989). In this paradigm, a stimulus-free interval separates the conditioned stimulus (e.g., an auditory tone) from the unconditioned stimulus (e.g., an air puff to an eye to induce a blink). Therefore, to bridge this temporal gap, neural activity elicited by the conditioned stimulus must be sustained throughout the interval without any further input. Vincent-Lamarre et al. (2016) demonstrated that providing a reservoir with external sinusoidal oscillations (from the oscillators shown at the top of Fig. 1) allows the reservoir to sustain their activity during an interval, allowing timing to be successfully learned. Kawai et al. (2025, 2024) improved performance by adding output feedback to the model and showed that it could learn and predict complex time series. In their model, the cerebellar reservoir receives oscillatory inputs from the hippocampus or other cortical areas. To date, however, reservoir models have not learned and generated real-world rhythms.

While most previous computational models have focused on rhythm perception, the current study shifts the focus to rhythm production. The perception models discussed below therefore provide a context for the current state of the field. Entrainment (Large and Jones 1999; Large et al. 2010, 2015) and error-correction (Mates 1994; Bose et al. 2019; Egger et al. 2020; Zemlianova et al. 2022) models have been proposed to explain the ability to perceive a beat from a computational perspective (for a review, see Large et al. (2023)). The entrainment models rely on oscillators that resonate and entrain their inputs. Entrainment refers to the synchronization of two or more oscillating systems as a result of an external forces or interactions between them. Beat perception is regarded as synchronizing an external rhythmic input with a preprepared oscillators. Classical error-correction models adjust the timing of the next finger tap to reduce the error between the current tap and the stimulus (Mates 1994). Bose et al. (2019) proposed an error-correction model in which a beat-generation oscillator learned the spiking phase and period of a stimulus neuron receiving an isochronous stimulus sequence. A comparator counts the number of spikes in the oscillator and stimulus neuron and adjusts the oscillator frequency according to the error between their counts. Egger et al. (2020) derived an error-correction model using a firing-rate neuron model. The neurons build an oscillator that activates a ramp function, and its value is reset when a certain threshold is exceeded. The ramping speed depends on an input parameter that controls the oscillator frequency. The input value was adjusted at each reset such that the oscillator frequency matched the time between successive stimuli. These models represent isochronous rhythms by tuning the frequency of an oscillator or the frequencies of oscillators. However, simple oscillators can only represent simple beats and do not generate complex rhythms.

Machine learning methods for generating music and complex rhythms are being actively developed (Briot and Pachet 2020; Ji et al. 2023). From reviewing the generation of drum performances, Hutchings (2017) proposed a method for generating a full drum kit part for a provided kick-drum sequence using an RNN sequence-to-sequence method. Makris et al. (2019) combined a long short-term memory- (LSTM) based architecture with a feedforward neural network module. The former module learns the sequences of successive drum events, whereas the latter deals with musical information on the guitar, bass, beat structure, and tempo. Thus, it can generate rhythms conditioned by musical information. Variational autoencoders (VAEs) learn to compress the input data into a latent distribution and reconstruct their input data (Kingma and Welling 2014). The decoder network generates a novel output from the latent space. GrooVAE (Gillick et al. 2019) automatically composes drum performances using a VAE, which generates and controls expressive drum performances. Furthermore, Transformers (Vaswani et al. 2017), which are a type of a deep learning model, have been applied in the field of music generations (Jin et al. 2020; Huang and Yang 2020; Nuttall et al. 2021). The learning algorithms of these machine learning systems (Briot and Pachet 2020; Ji et al. 2023; Hutchings 2017; Makris et al. 2019; Kingma and Welling 2014; Gillick et al. 2019; Vaswani et al. 2017; Jin et al. 2020; Huang and Yang 2020; Nuttall et al. 2021) are based on error back-propagation and do not aim to understand the brain processing. Their learning costs are very high, and they require large computational resources and training data. In contrast, reservoir computing is more biologically plausible because its learning algorithm does not rely on backpropagation and can be realized by simple rules, including LTD and dopaminergic modification in readout. Reservoir computing has fewer training parameters and can, therefore, learn from a small amount of data.

This study investigates the computational principles underlying the learning and generation of complex drum rhythms. Here, we define this complexity not by long-term metrical structures like syncopation, but rather by the subtle, micro-level fluctuations in timing intervals (microtiming) and sound intensity (amplitudes), and we focus on the acoustic features that constitute the rhythm’s groove sensation. To this end, we employ the reservoir model proposed by Kawai et al. (2025), a framework known as a timing-learning machine. The readout was trained with the desired target time series from the encoded performances of professional drummers. By feeding back the output of a reservoir to its input, the reservoir learns the next output from the current output; i.e., it predicts one time ahead. When generating rhythms, the reservoir repeats this one-time-ahead prediction and provides feedback to generate a time series. In standard reservoir models, the generated time series tends to be simple and periodic. To represent fluctuating timing intervals and amplitudes of skilled drumming performances, we input oscillations with multiple frequencies to the reservoir. It is expected that the input of oscillation superimposition produces more complex dynamics than the network-inherent dynamics, which facilitates the learning of acyclic drumming. Model outputs are then analyzed to estimate to what extent they mimic the original performances with respect to microtiming and audio features that related to a groove sensation. This study is the first to use reservoir computing to generate drum performances.

We hypothesize that oscillation-driven reservoir computing is a common computational principle underlying for learning of timing and rhythms in subcortical areas, such as the cerebellum and basal ganglia. Our model implements this hypothesis by mapping specific biological mechanisms to its components. Specifically, we model the synaptic plasticity essential for timing learning, such as LTD in the cerebellum and dopaminergic plasticity in the striatum, as the learning process for the reservoir’s readout weights. Furthermore, cortical oscillations provide the critical driving input to this subcortical reservoir. These temporally correlated but complex inputs enrich the reservoir’s internal dynamics, enabling it to produce non-isochronous rhythmic patterns rather than collapsing into simple isochronous rhythms. Consequently, our framework illustrates how the principal collaboration between cortical oscillations, as a source of fluctuations, and subcortical learning mechanisms gives rise to complex rhythmic patterns, including microtiming.

First, the model learns the one-dimensional hi-hat rhythm pattern by Jeff Porcaro. We tested the models with oscillations in different frequency bands and analyzed the nature of the generated time series. Using the same analyses as Räsänen et al. (2015), we examined whether the rhythms generated after learning were similar to the original performance. Although they used DFA to analyze long-range correlations in Jeff Porcaro’s hi-hat performance, we employed power spectral density (PSD) analysis instead, because DFA is susceptible to influence from periodic signals within the data (Nelias and Geisel 2024). Next, the model was trained to perform as a multidimensional drum kit. Microtiming and audio features of the outputs were analyzed and compared with those of the original performances. We present examples of the generated time series to demonstrate that the reservoir model can copy and generalize the performances in different genres.

## Methods

### Oscillation-driven reservoir computing

The reservoir is a randomly connected RNN with firing-rate neural units that receive multiple sinusoidal oscillator inputs (Fig. 1) (Kawai et al. 2025). The frequencies and phases of the oscillators are randomly determined, and once set, they are fixed across all trials. The initial states of the reservoirs are randomly determined. To reduce the initial state dependency, an onset signal is fed into the reservoir at time zero. This signal is a short, strong single pulse that resets the reservoir activity (Laje and Buonomano 2013; Kawai et al. 2023b). The output is obtained by the linear summation (readout) of the reservoir activity. The output is then fed back into the reservoir at the next time step. The readout weights are trained to minimize the errors between the outputs and target time series using recursive least squares (Haykin 2009), which is an online learning method. The other connection weights for the oscillator inputs, onset signal input, reservoir, and output feedback are determined randomly and fixed through learning.

The reservoir is a network with *N* neural units whose state vector is represented as ${\textbf{x}}(t) = (x_1, x_2, \dots , x_N)^\top$ at time *t*. The initial values of ${\textbf{x}}(t)$ are randomly drawn from a uniform distribution of $[-1, 1]$. Its network dynamics follows

1 $$\begin{aligned} \tau _\textrm{tc} \frac{\textrm{d}{\textbf{x}}(t)}{\textrm{d}t}= & -{\textbf{x}}(t) + {\textbf{W}} {\textbf{r}}(t) + {\textbf{W}}_\textrm{os} {\textbf{o}}(t) + {\textbf{W}}_\textrm{in} s(t) + {\textbf{W}}_\textrm{fb} {\textbf{y}}_\textrm{fb}(t), \end{aligned}$$

2 $$\begin{aligned} {\textbf{r}} (t)= & \tanh ({\textbf{x}}(t)), \end{aligned}$$

where $\tau _\textrm{tc}$ denotes the time constant, and ${\textbf{W}}$, ${\textbf{W}}_\textrm{os}$, ${\textbf{W}}_\textrm{in}$, and ${\textbf{W}}_\textrm{fb}$ denote the reservoir recurrent weight matrix, oscillator input weight matrix, onset signal input weight vector, and feedback weight matrix, respectively. The variables ${\textbf{o}}(t)$, *s*(*t*), and ${\textbf{y}}_\textrm{fb}(t)$ denote the oscillatory inputs, an onset signal, and the output feedback signals, respectively.

$${\textbf{W}}$$ is an $$N \times N$$ weight matrix, each component of which has a non-zero value with a probability *p*. The non-zero values are drawn from a Gaussian distribution with a mean of zero and a standard deviation (SD) of $$g/\sqrt{pN}$$, where *g* is the gain of the reservoir weights. The oscillators are $$N_\textrm{os}$$ sinusoids. The vector of the oscillator input is represented as $${\textbf{o}} (t) = (\sin (2 \pi f_1 t + \phi _1), \sin (2 \pi f_2 t + \phi _2), \dots , $$$$\sin (2 \pi f_{N_\textrm{os}} t + \phi _{N_\textrm{os}}))^\top $$, where $$f_i$$ and $$\phi _i$$ denote the frequency and the random initial phase, respectively. The frequencies are randomly determined from a uniform distribution of $$[f_\textrm{min}, f_\textrm{max}]$$ Hz. Parameters $$f_i$$ and $$\phi _i$$ are fixed during learning and testing. $${\textbf{W}}_\textrm{os}$$ is an $$N_\textrm{os} \times N$$ matrix for the oscillator input to the reservoir. The scalar onset signal *s*(*t*) is defined as 1 for the 50 ms time interval immediately preceding the onset (from $$t = -50$$ ms to $$t = 0$$ ms), and 0 at all other times. $${\textbf{W}}_\textrm{in}$$ is the weight vector of size *N* for the onset signal inputs into the reservoir. The output is fed back into all reservoir units as $${\textbf{y}}_\textrm{fb}(t)$$. The feedback weights $${\textbf{W}}_\textrm{fb}$$ is an $$N_\textrm{ro} \times N$$ matrix. The components of $${\textbf{W}}_\textrm{os}$$, $${\textbf{W}}_\textrm{in}$$, and $${\textbf{W}}_\textrm{fb}$$ are drawn from Gaussian distributions with a mean of zero and SDs of $$g_\textrm{os}/\sqrt{N_\textrm{os}}$$, $$g_\textrm{in}$$, and $$g_\textrm{fb} / \sqrt{N_{\textrm{ro}}}$$, where $$g_\textrm{os}$$, $$g_\textrm{in}$$, and $$g_\textrm{fb}$$ denote the gains of the oscillatory input weights, onset signal input weights, and feedback weights, respectively.

To obtain the *i*th output $y_i (t)$ ($i = 1, 2, \cdots , N_\textrm{ro}$), ${\textbf{r}}(t)$ is weighted as

3 $$\begin{aligned} y_i (t) = {\textbf{W}}_{\textrm{ro},i}(t) \mathbf {{\tilde{r}}}(t), \end{aligned}$$

where ${\textbf{W}}_{\textrm{ro},i}(t)$ denotes the *i*th $(3/2*N) \times 1$ readout weight vector, and $\mathbf {{\tilde{r}}}(t)$ is the concatenation of ${\textbf{r}}(t)$ and a vector of half components of ${\textbf{r}}(t)$ squared. By including these quadratic features, the readout layer itself can express nonlinear mappings from the reservoir dynamics to the target output, which enriches the model’s overall expressive power. This has been empirically shown to improve forecasting performance (Pathak et al. 2018; Chattopadhyay et al. 2020; Vlachas et al. 2020). ${\textbf{W}}_{\textrm{ro},i}(t)$ has an initial value for the zero matrix and is modulated by recursive least-squares, which is an online learning method. At time *t*, ${\textbf{W}}_{\textrm{ro}_i} (t)$ is updated as follows:

4 $$\begin{aligned} {\textbf{W}}_{\textrm{ro}_i} (t + \Delta t)= & {\textbf{W}}_{\textrm{ro}_i} (t) - e_i(t) {\textbf{P}}(t) {\textbf{r}}(t), \end{aligned}$$

5 $$\begin{aligned} e_i(t)= & y_i(t) - d_i(t), \end{aligned}$$

where $d_i(t)$, $e_i(t)$, and ${\textbf{P}}(t)$ denote the *i*th target, the *i*th error, and $(3/2*N) \times (3/2*N)$ matrix, respectively. ${\textbf{P}}(t)$ is updated as

6 $$\begin{aligned} {\textbf{P}}(t + \Delta t) = {\textbf{P}}(t) - \frac{{\textbf{P}}(t) {\textbf{r}}(t) {\textbf{r}}^\top (t) {\textbf{P}}(t)}{1 + {\textbf{r}}^\top (t) {\textbf{P}}(t) {\textbf{r}}(t)}. \end{aligned}$$

The initial value of ${\textbf{P}}(t)$ is given as $(1/a){\textbf{I}}$, where ${\textbf{I}}$ denotes the identity matrix, and *a* is a constant.

In the simulations, the numerical solutions to Eq. (1) were obtained using the Euler method, where the simulation step size $\Delta t$ was set to 1 ms. The recursive least-squares (Eqs. (4)–(6)) was applied once every two steps, and $\Delta t$ was set to 2 ms. The simulation started at $-250$ ms, and the training period was from 1 ms to 20,000 ms. The training was repeated ten times, and the performance was evaluated during the untrained testing period.

We used the parameter values listed in Table 1 in all the simulations. These parameters follow those in Kawai et al. (2025). To learn the hi-hat-only performance, we set $N_\textrm{ro} = 1$; to learn the funk performance, $N_\textrm{ro} = 3$; and to learn other drum-kit performances, $N_\textrm{ro} = 5$.

**Table 1:** Parameter settings

Parameter	Description	Value
*N*	Reservoir network size	5000
$N_\textrm{os}$	Number of oscillators	10
*g*	Gain of reservoir weights	1.2
$g_\textrm{os}$	Gain of oscillatory input weights	0.5
$g_\textrm{in}$	Gain of onset signal input weights	5
$g_\textrm{fb}$	Gain of feedback weights	3
*p*	Connection probability	0.1
$\tau$	Time constant	10 ms
*a*	Initial value for recursive least squares	1

### Power spectral density analysis

Although Räsänen et al. (2015) employed DFA to reveal the presence of long-range correlations in the hi-hat performance, DFA is known to be susceptible to bias from periodic components in a time series, which can lead to inaccurate estimates of the scaling exponent (Marković and Koch 2005; Nagarajan and Kavasseri 2005; Nelias and Geisel 2024). This presents a potential challenge for the analysis of musical rhythms, which are often characterized by periodicity. Therefore, we performed PSD analysis on the original and model-generated time series to evaluate the 1/*f*-like fluctuations. This approach is robust to the periodic components because it isolates them as distinct spectral peaks, allowing for a clearer characterization of the underlying aperiodic, power-law fluctuations, a practice well-established in musical time-series analysis (Voss and Clarke 1978; Nettheim 1992; Boon and Decroly 1995; Nelias and Geisel 2024). Nevertheless, a comparison using DFA is informative because it was employed in the key previous study (Räsänen et al. 2015); therefore, we present its methodology and results in Appendix A.

The procedure for our PSD estimation follows the approach of Nelias and Geisel (2024). We processed both the model-generated outputs (as described in Sect. “Encoding and decoding of drum performances”) and Jeff Porcaro’s hi-hat performance data (Räsänen et al. 2015) in the same manner. First, each performance was converted into a time series by quantizing it onto a grid of 1/16th of a quarter note. If a time slot contained a hit, its value was set to the hit’s amplitude; otherwise, it was set to zero. This process resulted in a time series, denoted as *q*(*t*), where *t* is the discrete time index with a 64th-note resolution. Finally, to remove the DC component, we subtracted the mean of the series, $\langle q \rangle$, from *q*(*t*) to obtain the final signal $\Delta q(t) = q(t) - \langle q \rangle$.

The PSD of $\Delta q(t)$ was estimated using the multitaper method (Thomson 1982). A key parameter for this method is the time-bandwidth product (*NW*), where *N* and *W* denote the length of the time series and the bandwidth, respectively. This product governs the trade-off between the variance of the spectral estimate and its frequency resolution. After exploring several values, we set *NW* to 4. For the estimation, we used *pmtm* function in MATLAB. In this study, the simulation was run for 20 trials with different random seeds. To estimate a representative PSD spectrum, we averaged the spectra across these 20 trials for each model condition.

We then fitted the averaged PSD spectrum on a log-log scale with either a single linear or a piecewise linear model with two segments to characterize its power-law behavior. Since the frequency resolution of the multitaper method is approximately *W*, the regression was restricted to frequencies $f > W$ where the spectral estimate is considered reliable. The fitting was performed using a weighted least-squares method. This weighting is crucial to counteract the bias introduced by the increasing density of data points at higher frequencies in a logarithmic plot. Specifically, the weights were set proportional to the frequency spacing on the log-frequency axis, ensuring that each frequency decade contributes equally to the fit. To select the more appropriate model, we compared the single linear and piecewise linear fits using the adjusted $R^2$, defined as:

7 $$\begin{aligned} \textrm{adjusted}\; R^2 = 1 - \frac{(1 - R^2)(N - 1)}{N - \theta - 1}, \end{aligned}$$

where $R^2$ denotes the coefficient of determination calculated from the weighted residuals, and $\theta$ is the number of model parameters: $\theta =2$ for the single linear fit and $\theta =4$ for the piecewise linear fit. The slope of the single linear model is denoted by $\alpha$. For the piecewise model, the slopes of the lower and higher frequency segments are denoted by $\alpha _1$ and $\alpha _2$, respectively.

Finally, we constructed the 95% confidence intervals for the PSD estimate itself. Following standard multitaper theory, the spectral estimate at each frequency point is assumed to follow a scaled chi-squared ($\chi ^2$) distribution. With a time-bandwidth product of $NW = 4$, we used $K = 2NW - 1 = 7$ tapers, resulting in $2K = 14$ degrees of freedom. The 95% confidence interval for the true PSD value at a given frequency is therefore calculated based on the estimated PSD and the critical values of the $\chi ^2(14)$ distribution. The lower and upper bounds of the confidence interval were then determined by scaling the fitted lines using the critical values corresponding to the 2.5th and 97.5th percentiles of the $\chi ^2(14)$ distribution.

### Encoding and decoding of drum performances

The input to the model comprises three signals: an onset signal *s*(*t*), a set of external oscillatory signals ${\textbf{o}} (t)$, and a feedback signal from the model’s own output ${\textbf{y}}_\textrm{fb}(t)$. During training, the model’s readout is modified to minimize the error between its output and a target time-series ${\textbf{d}} (t)$, which encodes a professional drum performance. Once trained, the model is capable of generating time-series that exhibit statistical and temporal properties similar to the target data, without requiring the target signal as a guide. This section describes the encoding and decoding methods for the drum performance data.

**Fig. 2:**
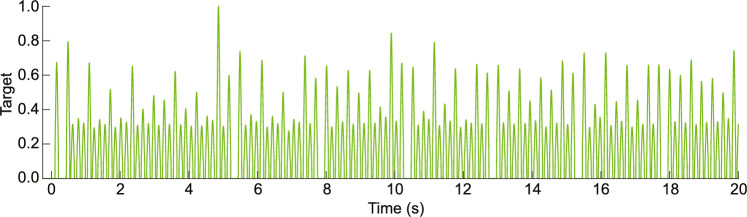
Target 20-s time series obtained from the hi-hat performance

The target time series was encoded from the hi-hat performance by Jeff Porcaro in “I Keep Forgettin’ ” by Mcdonald et al. (1982). We used the dataset of hi-hat hit timings and their amplitudes (Räsänen et al. 2015). This data was obtained by applying a 100th-order FIR filter with a cutoff frequency of 8 kHz to the original sound source to extract only the hi-hat sound and then applying an onset detection algorithm (Räsänen et al. 2015). The timing data were extracted with a precision of 1 ms. This level of temporal resolution is substantially finer than that required for the PSD analysis, which focuses on periodicities down to approximately 79 ms (12.7 Hz). To encode this data into the target time series, we first added 0.2 to all amplitudes to emphasize small amplitude hits. Single Gaussian pulses centered on the hit timing were then placed on a time series. The Gaussian amplitudes were the combined amplitudes of the hits, and their SDs were set to 30 ms. We cut out the first 20 s of the beginning and normalized it for the maximum and minimum to be 1 and 0 (Fig. 2). These hi-hat performance data, which consist of 16th notes, have six 16th rests after 4 s, and bar breaks after these rests. The relevant audio file is attached as Supplementary Information S01_target_hihat.wav.

To convert the model output into sound data, a moving average filter was first applied to the output time series, and a maximum value detection was performed to obtain hit timings (*u*(*i*)) and their amplitudes (*A*(*i*)). The amplitudes were renormalized such that the maximum and minimum amplitudes were those of the original. Hits with amplitudes below 0.1 were ignored. For subsequent analyses, this event series was also used to calculate the inter-beat intervals as $\tau (i) = u(i+1) - u(i)$. Finally, the sound was synthesized by placing hi-hat audio data at the timing points *u*(*i*). The gain of each audio sample was linearly scaled according to its corresponding amplitude *A*(*i*) to control the loudness.

We used the Groove MIDI Dataset (Gillick et al. 2019) which is a large dataset comprising 1150 MIDI files of drum performances by ten professional drummers in various genres. From this dataset, we selected four performances.

- Funk: drummer1/session1/1_funk_80_beat_4-4.mid,
- Jazz: drummer1/session1/4_jazz-funk_116_beat_4-4.mid,
- Samba: drummer1/session1/28_latin-samba_116_beat_4-4.mid,
- Rock: drummer1/session3/1_rock-prog_125_beat_4-4.mid.

Their MIDI files distinguishes between different types of drumming, e.g., between an open or closed hi-hat; however, hi-hats, snares, toms, and cymbals were grouped into one dimension each owing to the infrequency of hits. This procedure reduced the numbers of dimensions (pitches) of funk, jazz, samba, and rock from seven to three, from 12 to five, from six to five, and from six to five, respectively. The hit onset timings’ and their amplitudes’ data were extracted from the MIDI files and encoded into time-series data, as in the case of the hi-hat detailed above. A readout layer was provided for each multiple-drum instrument. The audio files of the targets are attached as Supplementary Information S02_target_funk.wav to S05_target_rock.wav.

Similar to above, the model outputs were converted to data of peak timings and their amplitudes. Fluctuation in the peaks resulted in small hits with small timing intervals to appear. Therefore, hits with timing intervals of 5 ms or less were removed as noise. The data were converted to the MIDI format and then converted to audio data.

In the audio analysis, we used Musical Information Retrieval (MIR) Toolbox for MATLAB (Lartillot and Toiviainen 2007). The MIR Toolbox functions we used for the analysis of the six features are listed below.

- Root mean square (RMS) energy (*mirrms*): global signal intensity (RMS of the amplitude).
- Spectral flux: averaged over the entire period of a spectral flux time series as the output of *mirflux*.
- Attack (*mirpulseclarity* using ‘Attack’ option): mean attack (amplitude) slope of all hit onsets.
- Pulse clarity (*mirpulseclarity* using ‘MaxAutocor’ option): clarity of rhythmic or metrical pulsation.
- Event density (*mireventdensity*): the number of events per second.
- RMS SD: SD over the entire period of an RMS time series as the output of *mirrms*.

## Results

### Learning the hi-hat performance

**Fig. 3:**
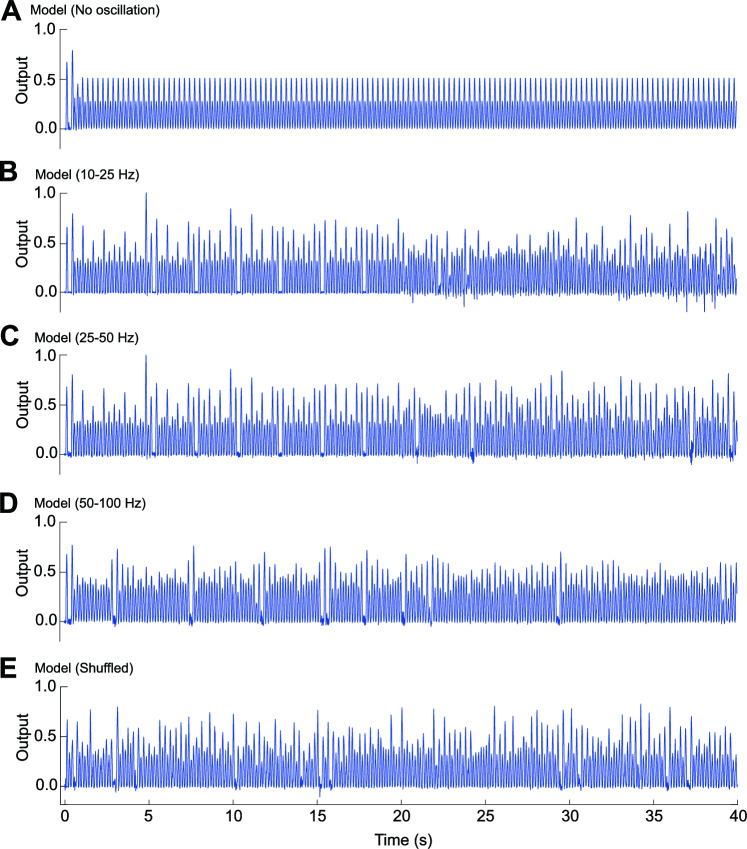
Example outputs of the reservoir model trained with the hi-hat time series. **A** Output of the reservoir model without oscillatory inputs. **B–D** Outputs of the reservoir models with [10, 25] Hz, [25, 50] Hz, and [50, 100] Hz, oscillatory input ranges, respectively. **E** Output of the reservoir model with shuffled oscillatory inputs

The reservoir computing model was trained with the target 20-s time series and generated 40-s outputs. During the generation, the model was not given the target and generated outputs autonomously. The latter 20 s, in particular, are an inexperienced period during which the model is expected to produce outputs analogous to the target; that is, it generalizes the target. Figure 3A shows an example of the output generated by the reservoir model without oscillatory inputs. The output fell into a completely periodic orbit and did not have the rests and fluctuations characteristic of the original performance. The audio file of the example output shown in Fig. 3A is attached as Supplementary Information S06_without_hihat.wav.

In contrast to its periodic output, oscillations in the reservoir enabled it to reproduce and generalize the target (Fig. 3B–D). The behavior of the outputs depended on the frequency bands of the oscillations. When oscillations of [10, 25] Hz were given to the reservoir (Fig. 3B), the model reproduced the target during the first 20 s whereas the output was disturbed and lacked generalization ability in the second half. Figure 3C shows the output of the model with [25, 50] Hz oscillators. In the first half, the model followed the target; in the second half, the output was analogous to the target. When further high-frequency oscillations ([50, 100] Hz) were applied to the reservoir, the model did not replicate the target perfectly but produced an output similar to that of the target (Fig. 3D). The respective audio files are attached as Supplementary Information S07_10-25_hihat.wav to S09_50-100_hihat.wav.

To clarify the effect of the oscillatory inputs, we established a control condition using shuffled inputs for comparison. In this condition, the time series $o_i(t) = \sin (2 \pi f_i t + \phi _i)$ from each of the $N_\textrm{os}$ oscillators was randomly shuffled along its time axis in each trial. This process preserved the intensity distribution of the original oscillatory inputs but destroyed their temporal correlations, rendering them akin to white noise. As shown in Fig. 3E, the model fed with these shuffled inputs also produced un unsteady, complex time series that superficially resembled the target performance. The respective audio file is attached as Supplementary Information S10_shuffled_hihat.wav.

We quantified the similarity between the model-generated outputs and the original (target) data regarding fluctuations and patterns of inter-beat timing intervals and amplitudes. In the analyses, the original data for all times of one song were used. While all the amplitude data were analyzed, only the 16th note intervals were analyzed. Therefore, only the timing interval data between 100 ms and 200 ms were analyzed. The model generated a 200-s output, the first 20 s of which were discarded, and the remaining 180-s portion was included in the analyses. The results of these analyses were averaged among the outputs of 20 models with different random seeds.

**Fig. 4:**
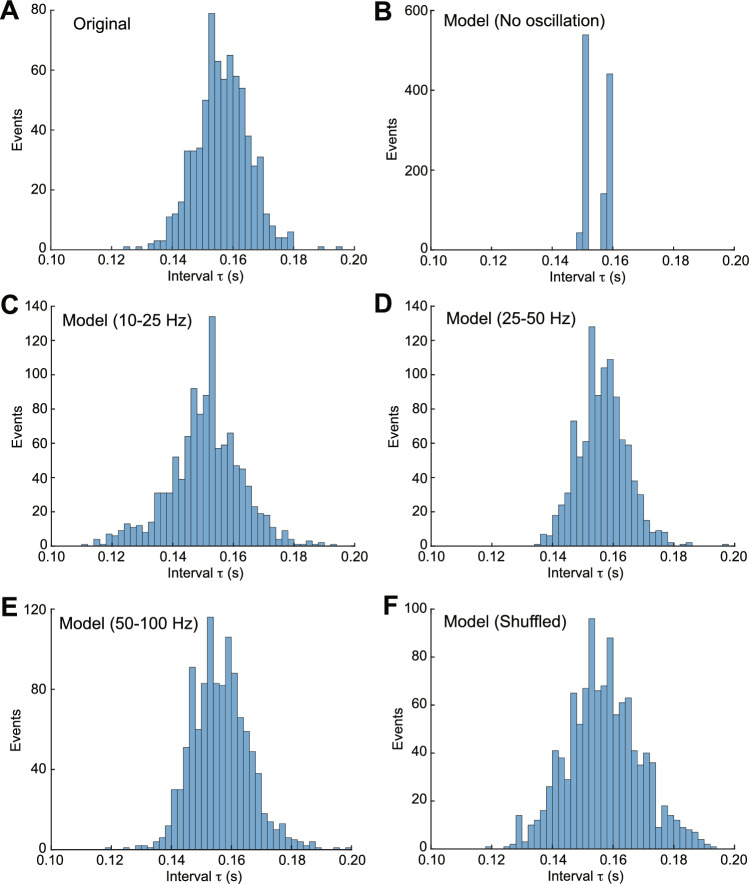
Histograms of hi-hat timing intervals $\tau (i)$. **A** the original performance data. **B** The model output without input oscillators. **C–E** the model outputs with oscillators in the frequency ranges of [10, 25] Hz, [25, 50] Hz, and [50, 100] Hz, respectively. **F** The model output with shuffled inputs. The histograms for the high-frequency input models (**D**, **E**) exhibit Gaussian-like distributions that resemble that of the original data (**A**)

Histograms of the timing intervals of the hi-hat hits are shown in Fig. 4. The timing intervals of the original data followed a Gaussian-type distribution, with a mean of 156.6 ms and an SD of 8.7 ms (Fig. 4A). In contrast, the output of the model without oscillators showed minimal fluctuation, with timing intervals at two discrete values (Fig. 4B). Introducing low-frequency oscillations ([10, 25] Hz) induced temporal variability, but the resulting distribution was non-Gaussian and characterized by heavier tails (Fig. 4C). Conversely, the models incorporating high-frequency oscillators ([25, 50] Hz and [50, 100] Hz) successfully generated interval distributions that resembled a Gaussian shape, similar to the original data (Fig. 4D and E). These results demonstrate that the high-frequency model could replicate the timing fluctuations of the original performance, known as microtiming. The histogram of the output timing intervals from the shuffled-input model exhibits a broader distribution than those from the models with oscillatory inputs and original data (Fig. 4F). The mean and SD of the timing intervals, obtained from 20 runs for each condition, are summarized under the “$\tau$ (ms)” column in Table 2.

To quantify the similarity between the distributions of timing intervals, $\tau (i)$, from the original performance and that from the model’s outputs, we computed the Wasserstein 1-distance (${\mathcal {W}}_1$). The Wasserstein distances, ranging from 0 to 1, measure the distance between two distributions, where a value closer to 0 indicates greater similarity. Table 2 presents the ${\mathcal {W}}_1$ for $\tau$ calculated between the original data and the outputs for each model condition, which was averaged across 20 runs. The results show that the high-frequency models ([25, 50] Hz and [50, 100] Hz) achieved smaller ${\mathcal {W}}_1$ values than the low-frequency model ([10, 25] Hz), the shuffled-input model, and the model without oscillators.

**Table 2:** Metrics of the original and model outputs. Mean (SD)

Source	$\tau$ (ms)	${\mathcal {W}}_1 (\tau )$ $\times 10^{-3}$	${\mathcal {W}}_1 (A)$ $\times 10^{-1}$	*r*
Original	157 (8.7)	-	-	$-0.48$
No oscillation	155 (5.3)	4.35 (1.12)	1.09 (0.076)	$-0.75$ (0.27)
10–25 Hz	151 (15.1)	6.65 (1.82)	1.13 (0.149)	$-0.075$ (0.057)
25–50 Hz	156 (8.6)	1.29 (0.39)	0.97 (0.057)	$-0.34$ (0.087)
50–100 Hz	155 (10.2)	2.09 (1.33)	0.88 (0.093)	$-0.48$ (0.14)
Shuffled	155 (11.8)	2.65 (1.62)	0.73 (0.060)	$-0.33$ (0.29)

**Fig. 5:**
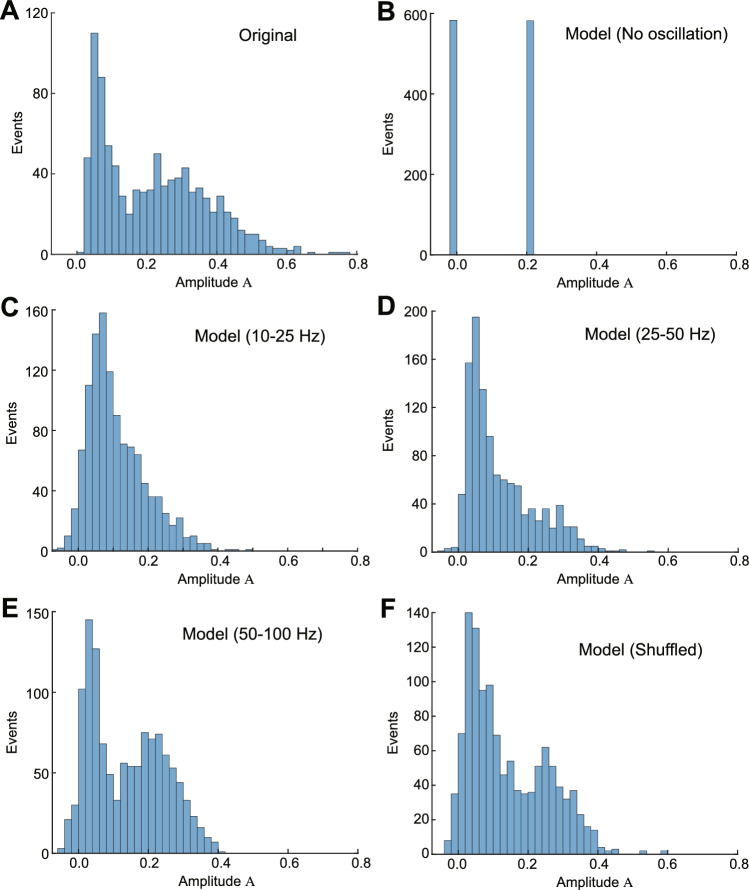
Histograms of hi-hat amplitudes *A*(*i*). **A** The original performance data. **B** The model output without input oscillators. **C–E** The model outputs with oscillators in the frequency ranges of [10, 25] Hz, [25, 50] Hz, and [50, 100] Hz, respectively. **F** the model output with shuffled inputs. The high-frequency (**E**) and shuffled-input models (**F**) both produce a bimodal distribution consistent with the original data (**A**)

Figure 5 shows amplitude histograms of the hi-hat hits. The original performance data displayed a bimodal distribution, reflecting a natural pattern of strong and weak accents (Fig. 5A). In contrast, the model without oscillators produced amplitudes concentrated at two discrete values, resulting in two sharp peaks and a lack of variability (Fig. 5B). The models with low- to mid-frequency oscillators ([10, 25] Hz and [25, 50] Hz) generated unimodal amplitude distributions that were skewed towards larger amplitudes (Fig. 5C and D), failing to reproduce the accent structure. Conversely, the high-frequency model ([50, 100] Hz) produced a bimodal amplitude distribution with significant variability, closely resembling that of the original performance (Fig. 5E). Similarly, the histogram of output amplitudes from the shuffled-input model also shows a clear bimodal distribution (Fig. 5F). These results indicate that the high-frequency model and shuffled-input model successfully captured both the characteristic strong-weak accentuation and its variability as observed in the original performance.

Table 2 summarizes the ${\mathcal {W}}_1 (A)$ values between the amplitude distribution of the original data and those of the model outputs. The high-frequency model ([50, 100] Hz) and the shuffled-input model exhibited the smallest ${\mathcal {W}}_1 (A)$ values. This result reflects their ability to reproduce the distinct bimodal amplitude distribution observed in the original data.

**Fig. 6:**
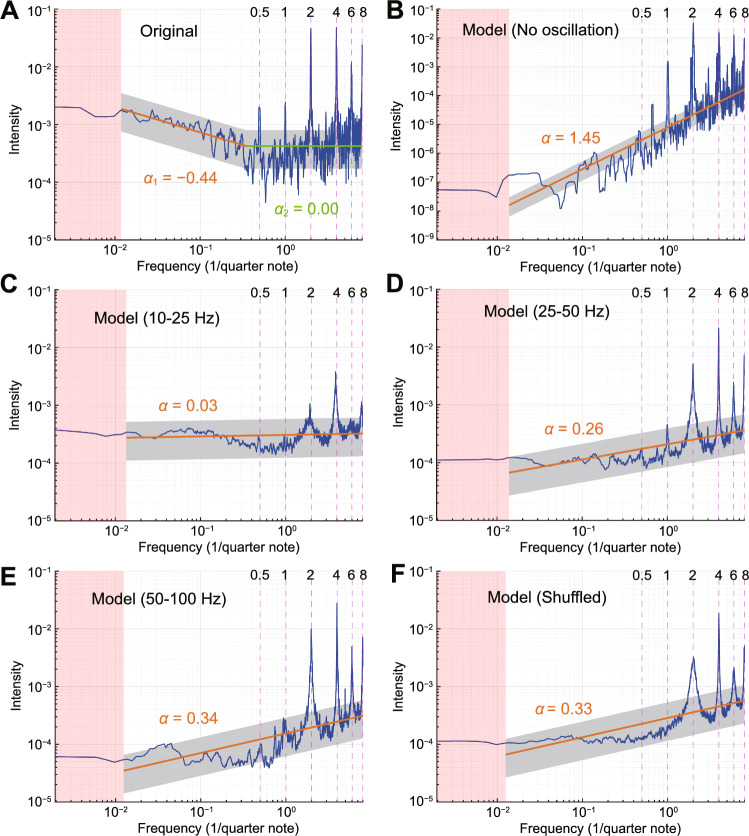
Results from PSD analysis. The grey shading indicates the 95% confidence interval for the PSD, with the orange and green segments representing the fitted (piecewise) linear model. The pink shading indicates frequencies below the bandwidth *W*. The magenta vertical broken lines indicate the peak frequencies observed in the original performance data. **A** The original performance data. **B** The model output without input oscillators. **C–E** The model outputs with oscillators in the frequency ranges of [10, 25] Hz, [25, 50] Hz, and [50, 100] Hz, respectively. **F** The model output with shuffled inputs. The power spectrum of the original data (**A**) shows both high-frequency peaks and a low-frequency 1/*f* trend. While the high-frequency models (**D**, **E**) successfully reproduce the high-frequency peaks, they fail to capture the low-frequency 1/*f* behavior

Second, we present the results of the PSD analysis. Figure 6A shows the PSD spectrum of the original performance, averaged across 20 trials. The horizontal axis represents frequency in units of cycles per quarter note. For context, at a tempo where one 16th-note interval is 0.157 s, a frequency of 1 cycle/quarter note corresponds to approximately 1.59 Hz (a period of 0.628 s). The PSD spectrum is characterized by two distinct linear segments in the log-log plot, with a breakpoint at approximately 0.34 cycles/quarter note. In the high-frequency region above the breakpoint, the spectrum exhibits six prominent peaks (indicated by magenta dashed lines) at 0.5, 1, 2, 4, and 8 cycles/quarter note, revealing a rich periodic structure. The strongest peak at 2 cycles/quarter note corresponds to an 8th-note rhythm (a period of two 16th-note steps), while the peak at 0.5 corresponds to a half-note rhythm. Away from these peaks, the spectral power forms high-frequency aperiodic fluctuations. In the low-frequency region below the breakpoint, the spectrum shows power-law behavior. The fitted slope, $\alpha _1$, was $-0.44$, indicating a $1/f^{0.44}$ dependency. This value, intermediate between white noise ($\alpha _1=0$) and 1/*f* pink noise ($\alpha _1=-1$), signifies the presence of weak long-range correlations, suggesting that fluctuations in past hits influence those in the future.

Figure 6B–F show the PSD spectra for the outputs of the model under various conditions. A key common finding is that none of the model-generated outputs exhibited the low-frequency power-law behavior observed in the original performance data. Instead, their low-frequency spectra were either flat or had a slight positive slope, indicating a lack of long-range correlations. However, the models reproduced the high-frequency periodic structures and aperiodic fluctuations. The model without oscillators (Fig. 6B) generated outputs that were mostly periodic, with relatively low-power fluctuations. Although the averaged PSD shows broad peaks, individual runs typically produced sharp peaks at a few discrete, run-specific frequencies. The model driven by low-frequency ([10, 25] Hz) oscillators showed weak peaks only at 2, 4, and 8 cycles/quarter note (Fig. 6C). The limited number of peaks and their lower amplitudes compared to the original data suggest the model generated only a simple periodic structure accompanied by noisy fluctuations. In contrast, increasing the oscillators’ frequency range led to richer rhythmic structures. The model with [25, 50]-Hz oscillators (Fig. 6D) produced stronger and more numerous peaks at 1, 2, 4, 6, and 8 cycles/quarter note, indicating clearer, richer periodic structures. This trend continued with the [50, 100]-Hz oscillator model (Fig. 6E), which additionally exhibited a weak peak at 0.5 cycles/quarter note, demonstrating its ability to generate a lower-frequency, half-note rhythm. The control model using shuffled inputs (Fig. 6F) produced peaks only at 2 cycles/quarter note and higher. This result confirms that without a temporally continuous oscillatory input, the model was unable to form temporal structures with periods on the scale of a quarter note or longer.

**Fig. 7:**
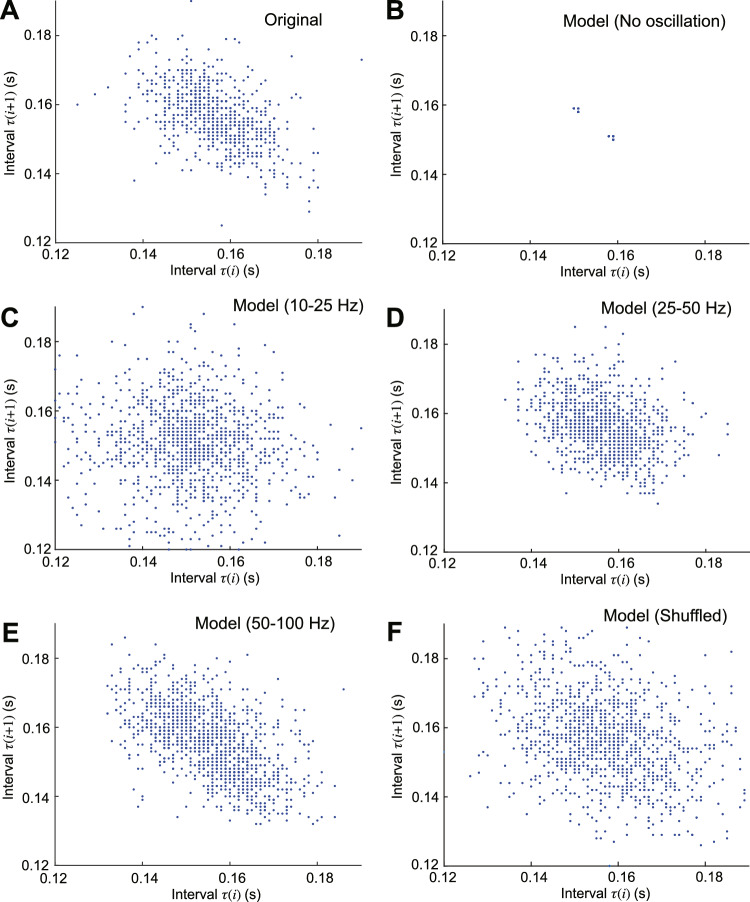
Return map of timing intervals $\tau (i)$. **A** The original performance data. **B** The model output without oscillators. **C–E** The model outputs with oscillators in the frequency ranges of [10, 25] Hz, [25, 50] Hz, and [50, 100] Hz, respectively. **F** The model output with shuffled inputs. The high-frequency model (**E**) successfully reproduces the negative correlation ($r = -0.48$) found in the return map of the original data (**A**), indicating its ability to capture short-term dependencies

Third, we analyzed the temporal dynamics of the timing intervals using return maps (Fig. 7). These maps are scatter plots created by plotting each timing interval $\tau (i)$ against the subsequent one ($\tau (i+1)$). The average correlation coefficients (*r*) from these plots over 20 runs are summarized in Table 2. The return map of the original performance data exhibited a negative correlation ($r = -0.48$), indicating a tendency for a shorter interval to be followed by a longer one, and vice versa (Fig. 7A). For the model without oscillators, the points formed two distinct clusters, revealing an alternating pattern between two specific timing intervals (Fig. 7B). In contrast, introducing low-frequency inputs to the model resulted in an uncorrelated plot (Fig. 7C). Notably, the correlation became progressively more negative as the input frequencies increased (Fig. 7D and E). While the high-frequency oscillator model ([50, 100] Hz) successfully replicated the negative correlation observed in the original data ($r = -0.48$), this correlation was weakened in the return map of the shuffled-input model ($r = -0.33$; Fig. 7F) This demonstrates that the temporal structure of the inputs is crucial for generating these one-to-one interval correlations.

**Fig. 8:**
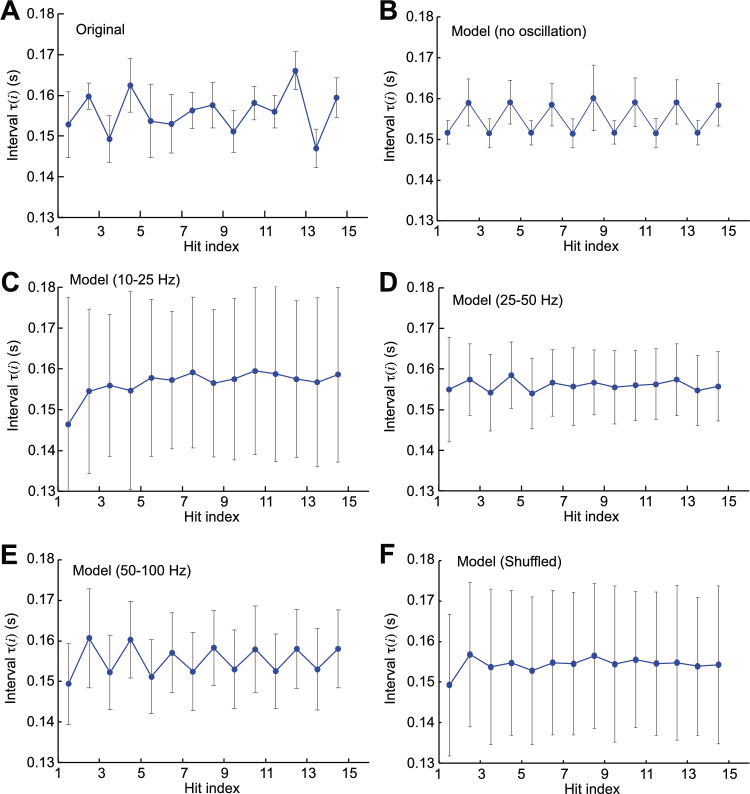
Bar patterns of timing intervals. **A** The first 10 bars of the original data. **B** The model output without input oscillators. **C–E** The model outputs with oscillators in the frequency ranges of [10, 25] Hz, [25, 50] Hz, and [50, 100] Hz, respectively. **F** The model output with shuffled inputs. Error bars indicate the SD. The high-frequency model (**E**) successfully reproduces the characteristic “short-long-short” pattern, a key bar structure present in the original data (**A**)

Finally, we analyzed the short-scale temporal patterns within the timing intervals $\tau (i)$, focusing on segments equivalent to bars consisting of 16 hits. Figure 8A shows the 16th-note intervals from the first 10 bars of the original performance data (the song’s introduction). A “short-long-short” pattern is observed at the beginning of the bars. Since the model outputs lacked a predefined bar structure, we applied different segmentation method. For the model without oscillators, we segmented the time series into non-overlapping, consecutive boxes of 16 data points to create artificial “bars,” starting the first box at an initial short timing interval. The averaged pattern across all such boxes, calculated over 20 runs, revealed a “short-long-short” sequence (Fig. 8B). For the models with oscillators, we defined bars as segments where a 16th rest was followed by 15 16th notes. The resulting patterns, averaged over 20 runs, showed that the low-frequency models ([10, 25] Hz and [25, 50] Hz) did not produce a clear or consistent pattern (Fig. 8C and D). In contrast, the high-frequency model ([50, 100] Hz) exhibited a clear “short-long-short” pattern following the 16th rests (Fig. 8E). This characteristic pattern was absent in the output of the shuffled-input model (Fig. 8F).

**Fig. 9:**
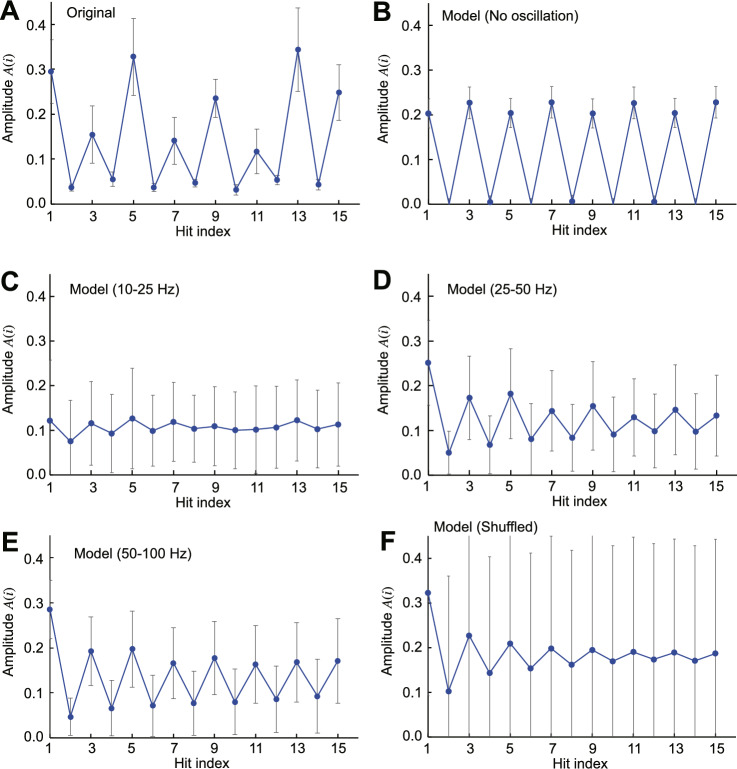
Bar patterns of amplitudes. **A** The first 10 bars of the original data. **B** The model output without input oscillators. **C–E** The model outputs with oscillators in the frequency ranges of [10, 25] Hz, [25, 50] Hz, and [50, 100] Hz, respectively. **F** The model output with shuffled inputs. Error bars indicate the SD. The high-frequency models (**D**, **E**) successfully capture the “strong-weak-strong” pattern characteristic of the original performance (**A**)

A similar analysis was conducted for the amplitude series *A*(*i*) (Fig. 9). The original performance data exhibited a distinct “strong-weak-strong” pattern (Fig. 9A). The model without oscillators clearly and consistently produced this “strong-weak-strong” amplitude pattern (Fig. 9B). In contrast, the outputs from the low-frequency model ([10, 25] Hz) lacked this structure (Fig. 9C). Although the high-frequency models ([25, 50] Hz and [50, 100] Hz) successfully generated the overall “strong-weak-strong” pattern (Fig. 9D, E), they failed to replicate the nuanced accentuation contour of the original performance. Specifically, while the models reproduced the pattern, the primary accents on the 1st, 5th, 9th, and 13th hits were less pronounced than in the original. In contrast, this “strong-weak-strong” pattern was both weak and highly variable in the output of the shuffled-input model (Fig. 9F).

### Learning drum-kit performances

**Fig. 10:**
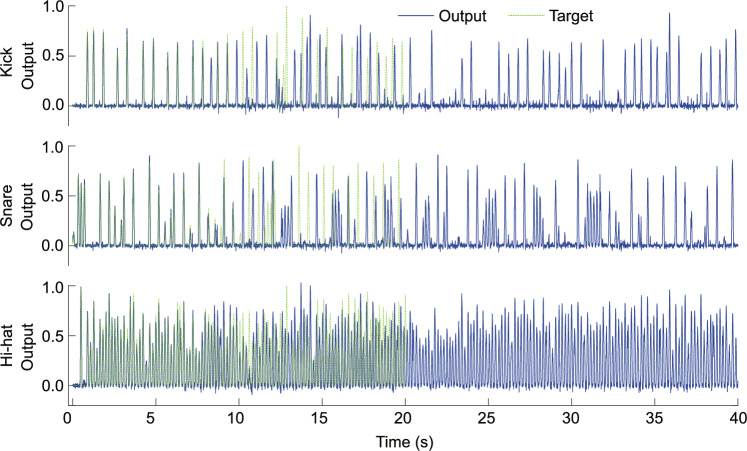
Example outputs of the reservoir model trained with the funk performance. The solid-blue and dashed-green curves represent the model’s output and the target, respectively

**Fig. 11:**
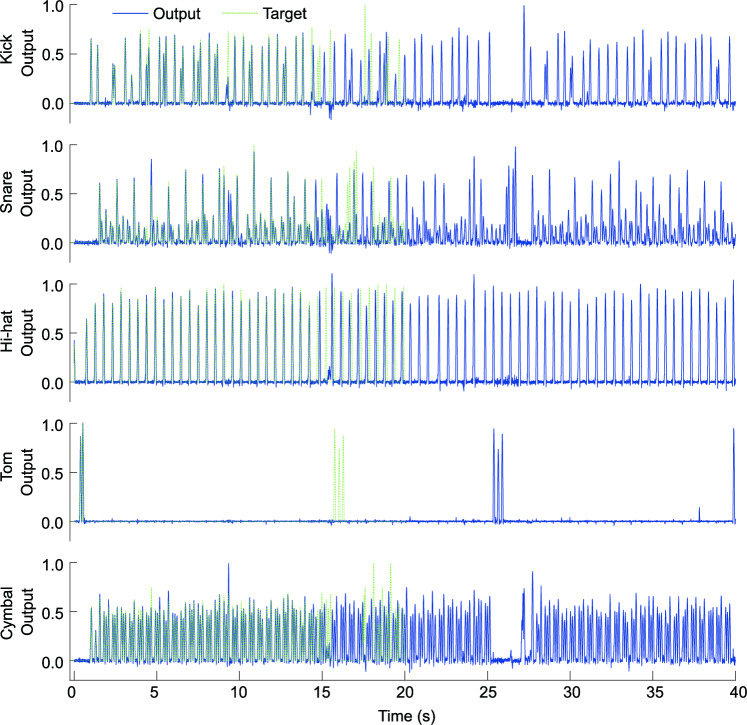
Example outputs of the reservoir model trained with the jazz performance. The solid-blue and dashed-green curves represent the model’s output and the target, respectively

**Fig. 12:**
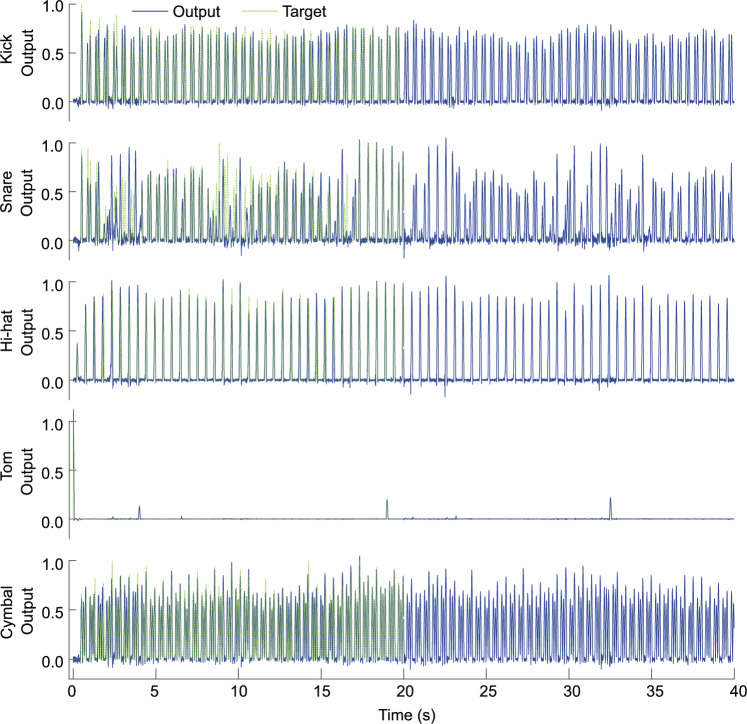
Example outputs of the reservoir model trained with the samba performance. The solid-blue and dashed-green curves represent the model’s output and the target, respectively

**Fig. 13:**
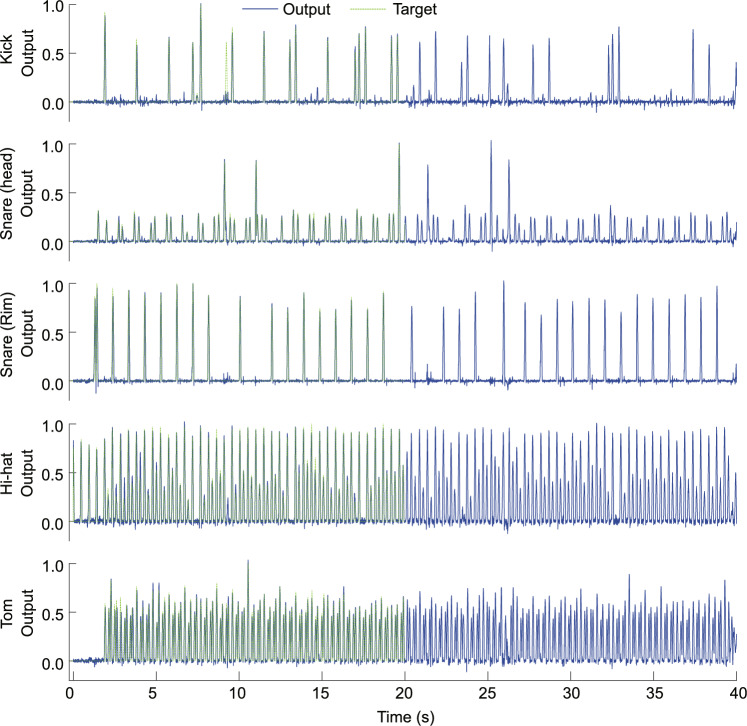
Example outputs of the reservoir model trained with the rock performance. The solid-blue and dashed-green curves represent the model’s output and the target, respectively

Next, the reservoir computing model with [50, 100]-Hz oscillators was trained using the target encoded from the multidimensional drum-kit performances from the Groove MIDI Dataset (Gillick et al. 2019). Figures 10–13 show example outputs of the model trained with the funk, jazz, samba, and rock performances, respectively. Their audio files are attached as Supplementary Information S11_output_funk.wav to S14_output_rock.wav. In each case, a performance similar to the target was generated. In the case of funk (Fig. 10), a series of snare hits emerged, which was not present. The jazz-learned model reproduced its characteristic fill-in (tom, snare, and kick (bass drum) patterns in that order), indicating the model learned such spatiotemporal pattern (Fig. 11). The samba output was relatively periodic, but the snare patterns dynamically transitioned (Fig. 12). In the case of rock (Fig. 13), a clear inter-modality rule was acquired that a snare (rim) was not struck when a snare (head) was hit with a large amplitude. These results demonstrate that the model could learn the coordination between drums to create dynamic, complex rhythmic patterns.

**Fig. 14:**
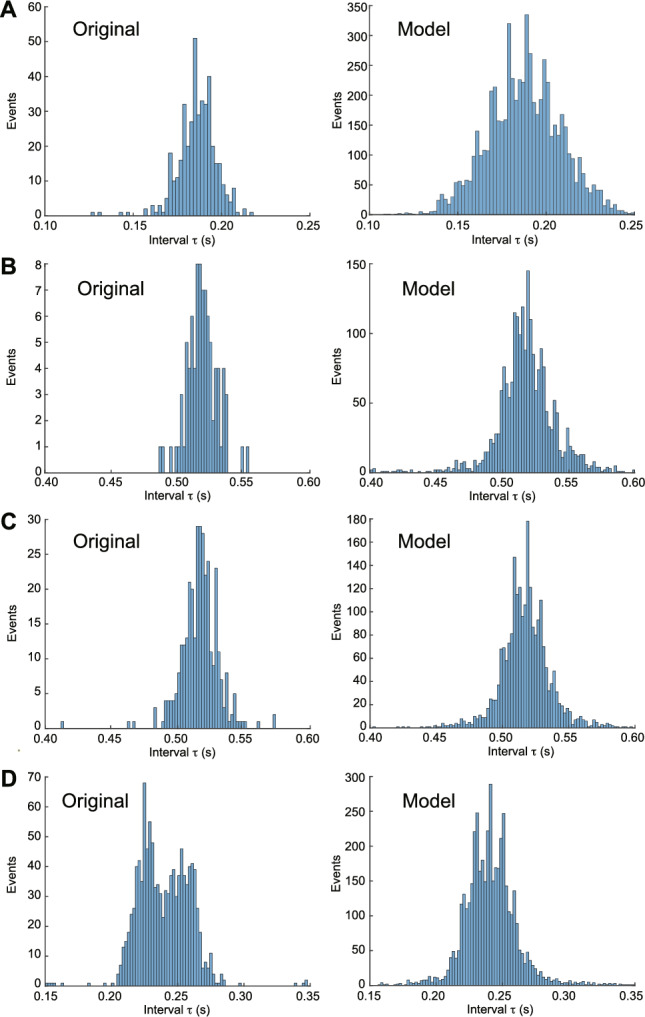
Histograms of the timing intervals of hi-hat hits in the four original performances (left panels) and model outputs (right panels). **A–D** Timing intervals for the funk, jazz, samba, and rock performances, respectively. While the models reproduce the mean inter-beat interval of the original data, their outputs exhibit greater variability (i.e., wider distributions) compared to the original performance

We investigated whether microtiming of the hi-hat performances in the model outputs reproduced that of the originals. The hi-hat rhythm was better suited for this microtiming analysis because it was more periodic than those of other drum parts. 20 models with different random seeds each output 60 s, and their hi-hat hit timing intervals were calculated. The hi-hat intervals for the entire original performances were also calculated. Figure 14 shows histograms of the hi-hat inter-beat intervals of the four original performances and model outputs. Histograms of the original performances showed bimodal peaks because the original hi-hat rhythm changed in the middle of the performances; therefore, in Fig. 14, the original histograms were cut out at the same intervals as the histograms of the model outputs. The means and SDs of those histograms are listed in Table 3. These results indicate that the average timing intervals matched between the originals and model outputs, whereas the microtiming variability of the model outputs was greater than that of the originals. This increased variability is a property of the model, as it was also observed in the previous hi-hat performance analysis (see Fig. 4 and the $\tau$ column in Table 2).

**Table 3:** Means and SDs of microtiming

Genre	Source	Mean (s)	SD (s)
Funk	Original	0.186	0.0108
	Model	0.188	0.0207
Jazz	Original	0.519	0.0115
	Model	0.518	0.0219
Samba	Original	0.517	0.0144
	Model	0.517	0.0187
Rock	Original	0.240	0.0194
	Model	0.241	0.0199

**Fig. 15:**
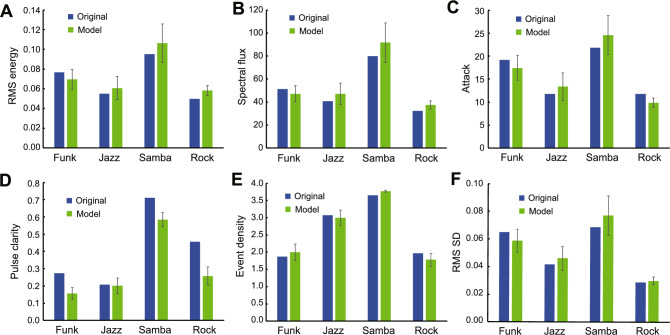
Audio features of the original performances (blue) and the model’s outputs (green). **A–F** Bar plots for RMS energy, spectral flux, attack, pulse clarity, event density, RMS SD, respectively. In model outputs, the error bars indicate the SDs among 20 models. The values for most audio features are consistent between the model outputs and the original data, with the notable exception of pulse clarity

The similarity of the audio features between the original performances and model outputs was quantified. We calculated six audio features related to groove feel (Stupacher et al. 2016) from the audio files of the original performances and the 20 model outputs. Specifically, these features were RMS energy, spectral flux, attack, pulse clarity, event density, and RMS SD, as detailed in Sect. “Encoding and decoding of drum performances”. The values of these audio features are shown in Fig. 15. We quantified the differences using the absolute percentage error, defined as the absolute difference between the model’s mean and the original value, normalized by the original value (Table 4). Overall, the results demonstrate a high degree of consistency for most features, indicating a acoustical similarity between the model outputs and the originals. However, for pulse clarity (Fig. 15D), the values of the model outputs were smaller than those of the originals. Since pulse clarity quantifies the periodicity of a rhythm, this result indicates that the periodicity or metric-structural components of the model outputs was reduced.

**Table 4:** Absolute percentage errors (%) between audio features of the original and model’s outputs

Audio feature	Funk	Jazz	Samba	Rock
RMS energy	9.1	10.3	11.8	24.0
Spectral flux	8.2	15.8	14.6	19.8
Attack	9.2	13.0	12.6	22.6
Pulse clarity	42.9	2.4	17.9	41.1
Event density	6.7	2.4	3.3	3.5
RMS SD	9.5	10.4	12.4	9.5

## Discussion

We developed a simple RNN model that learns complex drum performances using oscillation-driven reservoir computing. This model learned real-world drum performances and replicated the fluctuations of inter-beat timings (microtiming) and audio features relating to a sense of groove. Furthermore, it could generalize the short target data samples to generate rhythms that were close to but different from the target, which could be called improvisation based on an example. In the learning task of playing a multidimensional drum kit, the model could learn the spatiotemporal patterns between the instruments, resulting in dynamic and consistent performances, including fill-ins. Most existing models of beat perception and generation directly adjust the frequencies of neural oscillators to fit isochronous target rhythms (Mates 1994; Bose et al. 2019; Egger et al. 2020; Zemlianova et al. 2022). This approach allows only a simple rhythm to be generated. In the present model, the oscillator frequencies are fixed, and the network dynamics are tuned via readout training, which enables the model to learn complex real-world rhythms.

Oscillation-driven reservoir computing was designed for learning timings (Kawai et al. 2025). Time can be measured by reservoir activity driven by oscillator inputs, even during periods of no external stimulus. In this study, oscillators were used to generate complex reservoir activity. Conventional reservoir computing without oscillators produces simple periodic outputs. Although these outputs exhibited basic alternating patterns, such as a “short-long-short” pattern in timing interval series (Fig. 8B) and a “strong-weak-strong” pattern in amplitude series (Fig. 9B), they lacked the fluctuations characteristic of human performance. Consequently, applying the return map analysis to these outputs yielded no informative insights into such dynamics (Fig. 7B). In contrast, when driven by shuffled inputs, the reservoir produced timing and amplitude fluctuations whose overall statistical distributions mimicked those of the original performance data (Figs. 4F and 5F). The noisy inputs the simple periodic solutions of the reservoir, allowing it to generate more complex dynamics at a superficial level. However, a deeper analysis of the temporal structure revealed a critical divergence. The PSD analysis showed that the periodic structures in the shuffled model’s outputs were confined to short timescales (eighth-note scales or less; Fig. 6B). Furthermore, the pattern analysis confirmed a conspicuous absence of the long-range regularities that characterize the original performance, such as the “short-long-short” and “strong-weak-strong” patterns (Figs. 7F, 8F, and 9F). This finding highlights a clear dissociation between statistical mimicry and structural fidelity. While unstructured noise is sufficient for the reservoir to generate statistically plausible, moment-to-moment fluctuations, the structured oscillatory inputs are indispensable for scaffolding these fluctuations into the organized, long-range temporal patterns.

Driving the reservoir with high-frequency oscillators endowed it with the ability to generate sophisticated rhythmic structures that closely mirrored human performance. The model not only reproduced the key temporal patterns, such as “short-long-short” and “strong-weak-strong” (Figs. 7E, 8E and 9E), but also exhibited a rich hierarchy of periodicities spanning from short notes up to a half-note scale (Fig. 6E). However, a crucial distinction remained. Despite these similarities in deterministic structure, the model’s outputs failed to exhibit the 1/*f* power-law behavior in the low-frequency domain (Fig. 6A and E), a hallmark of the stochastic fluctuations found in the original human data. Unlike the unstructured shuffled inputs, which only drive variability, the oscillators offer the short-term rhythmic patterns. This suggests that the high-frequency oscillators provide a dynamic, internal scaffold upon which the reservoir can build rich rhythmical sequences.

Given its ability to generate complex rhythms including temporal features, i.e., local patterns and fluctuations, we propose this architecture as a unified model for learning timings and rhythms. In the human brain, the cerebellum and basal ganglia are thought to be responsible for such learning (Zatorre et al. 2007; Grahn and Rowe 2009; Teki et al. 2012; Grahn 2012; Fujii and Wan 2014; Merchant et al. 1664; Kasdan et al. 2022; Etani et al. 2024). Indeed, the fact that previous computational models in these areas also used a reservoir computing framework (Yamazaki and Tanaka 2007; Rössert et al. 2015; Tokuda et al. 2021; Dominey 1995; Hinaut and Dominey 2013; Kawai and Asada 2023) further supports our model.

Our choice of a reservoir computing framework in this study was not merely based on its precedent in computational neuroscience but was motivated by its computational capacity utilizing those neural dynamics. The rich, high-dimensional dynamics of a reservoir provide an ideal substrate for generating the complex and context-dependent temporal patterns characteristic of expert performance. Then, the readout selects the desired dynamics as its output. This is functionally analogous to the role of subcortical circuits like the basal ganglia, which are thought to select cortical dynamics for motor behavior (Gurney et al. 2001; Baladron et al. 2023). Furthermore, expressive timing critically requires prediction, a process that relies on maintaining a memory of the recent temporal context. The intrinsic “fading memory” of reservoir computing, where the reservoir’s state retains a trace of past inputs (Maass et al. 2002; Gonon and Ortega 2021), directly models this neural requirement to bridge temporal gaps and anticipate future events, a function often attributed to the cerebellum and basal ganglia. Finally, the learning paradigm of reservoir computing, which combines a fixed reservoir with a trainable readout layer, resonates with a biologically plausible learning scheme (e.g., plasticity in Purkinje cells and the striatum). It is this convergence of principles—rich dynamics for generation, temporal memory for prediction, and an efficient learning architecture—that establishes reservoir computing as a functionally compelling model for investigating the neural basis of timing and rhythm production.

The model’s performance depended on the given frequency bands. Low-frequency ([10, 25] Hz) inputs contributed to the accurate reproductions of the targets, whereas high-frequency ([50, 100] Hz) inputs contributed to generalizing the target to generate target-like performances. This tendency is similar to that observed in previous studies on chaotic time-series prediction (Kawai et al. 2025; Park et al. 2025). We hypothesize that the low-frequency inputs acted as “time stamps” for the targets. That is, the model learned the relationship between the specific dynamics induced by the oscillator inputs, which represented time, and the targets. However, the model that overfitted the relationship between specific reservoir states and the target was unable to generalize it to unlearned states. In contrast, high-frequency inputs did not significantly affect the reservoir states because the neuron model had a time constant and did not change states at high speed. Therefore, the model somewhat preserved the network-inherent dynamics, and thus prevented overfitting.

This suggests that different neural mechanisms are involved in copying and improvising musical performances. High-frequency (beta- and gamma-band) oscillations were observed in magnetoencephalography (MEG) during music beat perception, imagery, and timing prediction (Fujioka et al. 2009, 2015). An electroencephalogram (EEG) study by Rosen et al. (2020) found clusters of beta- and gamma-band activity when higher-quality and lower-quality improvisations were compared in jazz performances. Subsequently, a group of the authors (Rosen et al. 2024) recorded jazz guitarists’ EEG while improvising to provide chord sequences. They found that high-flow (effortless attention to the task) was associated with high-frequency (gamma-band) clusters in the left opercula and in the left temporal gyri. Such high-frequency cortical activity may facilitate the generalization of musical performances. Using EEGs to detect further high-frequency (high-gamma-band) signals is difficult, but studies using electrocorticography (ECoG) have revealed that rhythmic information is contained in the high-gamma signals in the auditory cortex (Sturm et al. 2014; Ding et al. 2019; Herff et al. 2020). This confirms that listening to music induces high-frequency signals, and it would be interesting to study how this relates to improvising performances.

The frequency-dependent division of labor—reproduction via low frequencies and generation via high frequencies—opens up an intriguing possibility for how the brain might flexibly control performance. It might be plausible that the brain could dynamically tune its sensitivity to different frequency bands depending on the task demands. For instance, a musician tasked with faithfully imitating a piece would benefit from prioritizing inputs in the lower-frequency bands to ensure accurate reproduction. Conversely, during improvisation, where the goal is to generate novel yet stylistically coherent patterns, the brain might up-weight inputs from higher-frequency bands to leverage their generative capabilities. Elucidating the neural mechanisms that enable such flexible switching is a challenge for future research.

In the present study, the specific frequency bands used were not directly related to the task of rhythm learning. For example, the average inter-beat intervals for the hi-hat performance is 0.156 s, which corresponds to approximately 6.4 Hz. The frequencies of the input oscillators were much higher than those and were randomly determined. Even though the model did not have an external “metronome,” it acquired an internal representation of a metronome as a result of training, resulting in stable performances.

In learning the hi-hat rhythm, the model generated performances with fluctuations in the timing intervals and amplitudes. These fluctuations were not random but exhibited the same distributions and patterns (e.g., the high-low-high-low pattern) as the original fluctuations. Such repetitive timing deviation is called *systematic* microtiming (Madison et al. 2011; Madison and Sioros 2014), and microtiming within the same instrument is considered *horizontal*. Therefore, the model could copy *systematic horizontal* microtiming of the professional hi-hat performance. However, the model’s outputs failed to exhibit the characteristiclow-frequency 1/*f* behavior, which is a limitation of this model. This discrepancy is likely attributable to the model’s failure to learn the long-range dependencies associated with bars and meters. The 16th rests, which marks the end of the bars in the original hi-hat performance, was randomly placed in the model’s outputs. Therefore, this model fails to learn the long-term rhythmic patterns. A solution may conceivably be to include meter information in the oscillators. An EEG study revealed that gamma-band signals in the auditory cortex reflect the metric structure (Snyder and Large 2005). Other studies have also shown that brain oscillations in various frequency bands contain information about the meters of rhythms being heard (Fujioka et al. 2009; Iversen et al. 2009; Nozaradan et al. 2011). By including meter information in the oscillations, it may be possible to learn long-term rhythmic patterns.

In learning the drum-kit performances of various genres, the model generated outputs similar to the original performances. The microtiming analysis of the hi-hat performances showed that the model outputs had horizontal microtiming with greater variability than the originals. This might be due to the variability in neural dynamics of the model. Nevertheless, their SDs were less than 20 ms, which are considered as microtiming, defined as timing deviation of 50 ms or less. The audio feature analysis showed that with except for the pulse clarity feature, the model outputs copied the features that correlate with a sense of groove, indicating that the model can copy groovy performances. The pulse clarity values for periodicity were reduced because the model did not represent long-term structures, including meter and bar, as discussed earlier.

The model presented here uses professional performance as a target and copies it in only ten training sessions. Therefore, it does not explain how the drumming performance improves over time through practice. In reality, performance skills are acquired gradually during numerous practice routines designed and arranged by teachers and coaches (Ericsson et al. 1993; Anders 2008). Performing music involves more than a single neural circuit but a variety of sensorimotor and cognitive functions (Brown et al. 2015), such as motor control (Aoki et al. 2005; Fujii et al. 2009), planning and temporal control (Drake and Palmer 2000), and feedback monitoring (Repp 1999). An important future research direction is to investigate the processes for acquiring such complex skills to construct their computational models.

## Conclusion

In this study, we investigated the capacity of an oscillation-driven reservoir computing model to learn and generate complex drum rhythms. Our findings demonstrate that the model with high-frequency inputs can successfully acquire and reproduce the subtle microtiming variations and expressive dynamics that are characteristic of professional drumming. This suggests that a relatively simple RNN architecture is sufficient to capture the fine-grained temporal patterns.

However, we identified a notable limitation: the model was unable to learn higher-level rhythmic structures, such as meter and bar divisions. While it could generate locally coherent patterns, it failed to adhere to the long-term, hierarchical organization that governs most musical rhythms. This distinction suggests that the cognitive and computational mechanisms for processing local, expressive timing may be distinct from those required for understanding abstract, metrical hierarchies. Future research should focus on developing models that can integrate these two levels of rhythmic processing. Such work will be crucial for building a more comprehensive computational theory of human rhythmic capability.

## Supplementary information

- S01_target_hihat.wav: Audio file of the hi-hat target.
- S02_target_funk.wav: Audio file of the funk target.
- S03_target_jazz.wav: Audio file of the jazz target.
- S04_target_samba.wav: Audio file of the samba target.
- S05_target_rock.wav: Audio file of the rock target.
- S06_without_hihat.wav: Audio file of an output of the model without oscillations.
- S07_10-25_hihat.wav: Audio file of an output of the model with [10, 25]-Hz oscillators.
- S08_25-50_hihat.wav: Audio file of an output of the model with [25, 50]-Hz oscillators.
- S09_50-100_hihat.wav: Audio file of an output of the model with [50, 100]-Hz oscillators.
- S10_shuffled_hihat.wav: Audio file of an output of the model with shuffled oscillatory inputs.
- S11_output_funk.wav: Audio file of a model output trained with the funk.
- S12_output_jazz.wav: Audio file of a model output trained with the jazz.
- S13_output_samba.wav: Audio file of a model output trained with the samba.
- S14_output_rock.wav: Audio file of a model output trained with the rock.

## Supplementary Information

Below is the link to the electronic supplementary material.

Supplementary file 1 (zip 53221 KB)

## Figures and Tables

**Fig. 16:**
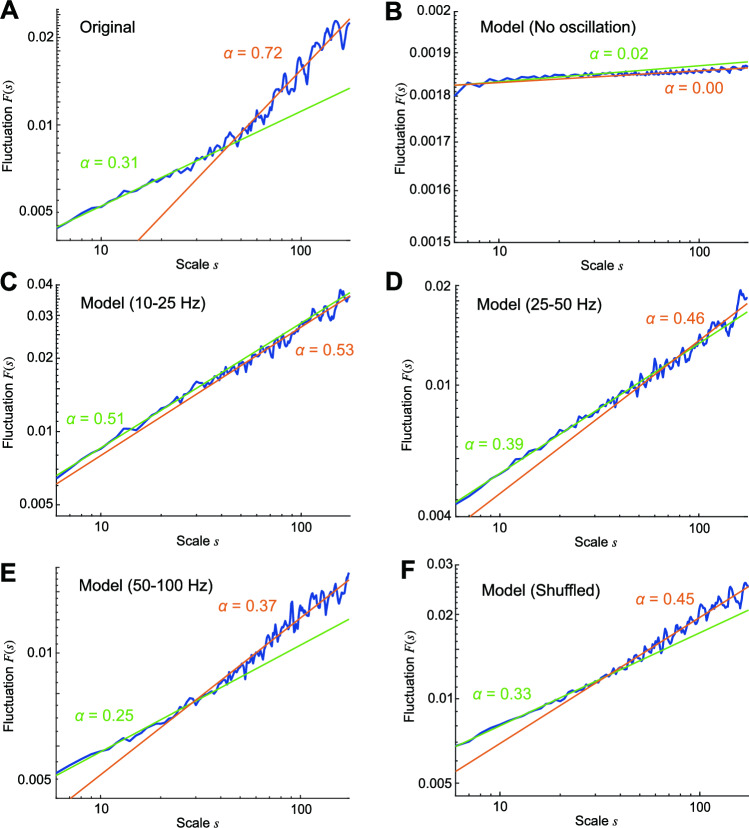
Results from DFA for timing intervals. **A** The original performance data. **B** The model output without input oscillators. **C–E** The model outputs with oscillators in the frequency ranges of [10, 25] Hz, [25, 50] Hz, and [50, 100] Hz, respectively. **F** The model output with shuffled inputs

**Fig. 17:**
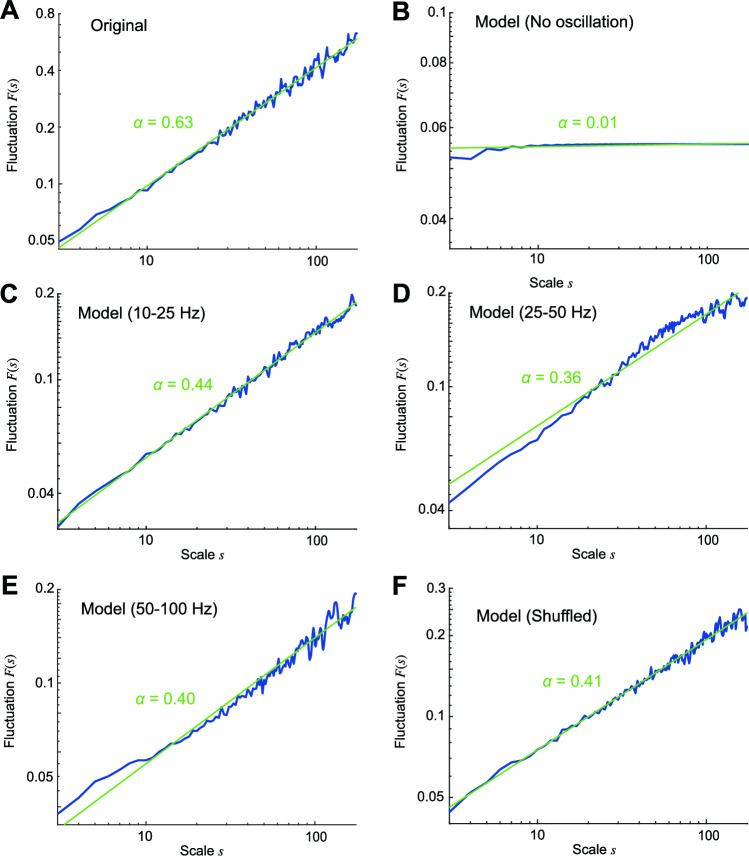
Results from DFA for amplitudes. **A** The original performance data. **B** The model output without input oscillators. **C–E** The model outputs with oscillators in the frequency ranges of [10, 25] Hz, [25, 50] Hz, and [50, 100] Hz, respectively. **F** The model output with shuffled inputs

## Data Availability

The data and code that support the findings of this study are openly available. The hi-hat timing data are derived from a publicly available dataset by Rasanen et al. (2015), accessible at https://journals.plos.org/plosone/article?id=10.1371/journal.pone.0127902. The Groove MIDI Dataset, as described in Gillick et al. (2019), is available at https://magenta.withgoogle.com/datasets/groove. The source code for our model is available on GitHub at https://github.com/Kawai-Yuji/ODRC_drum.

## Appendix A: Detrended fluctuation analysis (DFA)

DFA is a statistical method widely used to analyze the presence of long-range correlations in time-series data, particularly for non-stationary data, such as heartbeat time series (Peng et al. 1995), brain oscillations (Linkenkaer-Hansen et al. 2001; Parish et al. 2004), and human musical rhythms (Hennig et al. 2011; Räsänen et al. 2015). Long-range correlations refer to the persistence of dependencies between values that are far apart in a sequence. These correlations indicate that values separated by a long time period are still statistically related, which can indicate the underlying processes or structures driving the data. Räsänen et al. (2015) applied DFA to the inter-beat intervals and amplitudes of Jeff Porcaro’s hi-hat performance, reporting the presence of long-range correlations in both.

However, DFA is known to be sensitive to the periodic components that are inherent in musical rhythms (Marković and Koch 2005; Nagarajan and Kavasseri 2005; Nelias and Geisel 2024). It should be noted that the data in the previous study (Räsänen et al. 2015) were not continuous time-domain signals but rather event-based series, specifically sequences of inter-beat intervals and their amplitudes. Therefore, they do not directly reflect the explicit periodicity inherent in musical rhythms. However, we cannot preclude the possibility that these series still contain latent periodicity derived from the underlying musical structure (e.g., meter and measures), which could in turn bias or distort the DFA results. For this reason, the main body of this paper employed PSD analysis to evaluate the periodic structuresand the long-range (or 1/*f*-like) fluctuations in the original and model-generated performance data. Nevertheless, a comparison using DFA is informative, and this appendix presents its methodology and results to demonstrate the correspondence between the original performance and our model’s outputs.

### DFA procedure

We applied DFA to the inter-beat intervals and amplitudes. Let *u*(*i*) be the *i*th hit time, and the *i*th inter-beat interval be denoted by $\tau (i) = u(i+1) - u(i)$. To focus the analysis on 16th-note dynamics, any intervals longer than 200 ms were removed from the series. Then, we calculate the fluctuation of the timing interval from the mean $\langle \tau \rangle$, i.e., $\Delta \tau (i) = \tau (i) - \langle \tau \rangle$. The fluctuations are integrated as

A1 $$\begin{aligned} z(i) = \sum _{j=1}^i \Delta \tau (j) \;\; \textrm{for}\; i = 1, 2, \dots , M, \end{aligned}$$

where *M* is the number of data points. Next, the integrated time series is divided into boxes of equal length *s*. Within each box, a least-squares line ($z_s(i)$) is fitted to the data points (*y*(*i*)) to capture the linear trend. The trend represented by $z_s(i)$ was subtracted from the original data (*z*(*i*)) within the box, resulting in the detrended fluctuation: ($z_\textrm{detrended}(i) = z(i) - z_s(i)$). Finally, the root-mean-square fluctuations are calculated for each box by the following

A2 $$\begin{aligned} F_k (s) = \sqrt{\frac{1}{s} \sum _{i=ks+1}^{ks+s} \left[ z_\textrm{denrended} (i) \right] ^2} \;\; \textrm{for}\; k = 1, 2, \dots , \frac{M}{s} - 1. \end{aligned}$$

Finally, $F_k (s)$ is averaged over all *M*/*s* elements to obtain $F(s) = \langle F_k (s) \rangle$. This procedure is repeated for different box size *s*. In the presence of scaling, the relationship between *F*(*s*) and *s* may be described by $F(s) \propto s^\alpha$, where the scaling exponent $\alpha$ is the slope of the line relating $\log F(s)$ and $\log s$. Equation (A1) and below applied similarly to the amplitude data series.

The same procedure was applied to the ampltude series, where *A*(*i*) denotes the amplitude of the *i*th hit time. The amplitude series was normalized by the mean amplitude $\langle A \rangle$, i.e., $\Delta A(i) = A(i) - \langle A \rangle$, before applyin the above DFA procedure.

The scaling exponent $\alpha$ indicates the nature of the correlations; when $\alpha = 0.5$: the series is uncorrelated, e.g., for white noise; when $0.5< \alpha < 1$: the series has long-range positive correlations; when $0< \alpha < 0.5$: the series has long-range negative correlations.

### DFA results

DFA was applied to the timing intervals and amplitudes. Figure 16 shows the results of DFA for the timing interval fluctuations. For the original performance data, the DFA plot can be approximated by two lines bordered by a scale of 40 (Fig. 16A). Table 5 lists the corresponding exponents for the shorter and longer scales in the $\alpha _\tau$ (short) and $\alpha _\tau$ (long) columns. The exponent values for original data were 0.31 (anticorrelations) and 0.72 (correlations). In contrast, the exponent for the model without oscillators was approximately zero (Fig. 16B), reflecting that its timing interval series lacked significant fluctuations. The DFA plots for the models with low-frequency oscillators ([10, 25] Hz and [25, 50] Hz) did not show a clear separation between two scales (Fig. 16C and D). Their exponent values were approximately 0.5, which indicates that the fluctuations were uncorrelated, similar to white noise. The analysis of the high-frequency model ([50, 100] Hz) output yielded a DFA plot with two distinct slopes, similar to that of the original data (Fig. 16E). However, both of its exponents were less than 0.5, indicating the presence of anti-persistent correlations across both short and long scales. DFA applied to the output from the shuffled-input model also revealed a crossover between two scaling regimes; however, the exponent for the longer scale dropped below 0.5 (Fig. 16F).

**Table 5:** Mean DFA exponents of the original and model outputs. Mean (SD)

Source	DFA $\alpha _\tau$ (short)	DFA $\alpha _\tau$ (long)	DFA $\alpha _A$
Original	0.31	0.72	0.63
No oscillation	0.020 (0.025)	0.000 (0.002)	0.007 (0.004)
10–25 Hz	0.52 (0.033)	0.53 (0.092)	0.48 (0.044)
25–50 Hz	0.37 (0.068)	0.43 (0.088)	0.43 (0.066)
50–100 Hz	0.29 (0.068)	0.39 (0.13)	0.41 (0.085)
Shuffled	0.37 (0.057)	0.48 (0.16)	0.47 (0.15)

Unlike the timing intervals, the DFA for amplitude fluctuations exhibited a single, uniform scaling region across all conditions, as shown in Fig. 17. The corresponding scaling exponents ($\alpha _A$) are listed in Table 5. The original performance data yielded an exponent greater than 0.5 ($\alpha _A > 0.5$), indicating persistent, long-range correlations (Fig. 17A). In contrast, all model outputs that included oscillators produced exponents less than 0.5 ($\alpha _A < 0.5$), which is characteristic of anti-persistent correlations (Fig. 17C–F). This anti-persistence suggests a mean-reverting dynamic, where large amplitude values are likely to be followed by small ones, and vice versa.
